# Hyperspectral imaging for perioperative monitoring of microcirculatory tissue oxygenation and tissue water content in pancreatic surgery — an observational clinical pilot study

**DOI:** 10.1186/s13741-021-00211-6

**Published:** 2021-12-01

**Authors:** Maximilian Dietrich, Sebastian Marx, Maik von der Forst, Thomas Bruckner, Felix C. F. Schmitt, Mascha O. Fiedler, Felix Nickel, Alexander Studier-Fischer, Beat P. Müller-Stich, Thilo Hackert, Thorsten Brenner, Markus A. Weigand, Florian Uhle, Karsten Schmidt

**Affiliations:** 1 Department of Anesthesiology, Heidelberg University Hospital, Heidelberg, Germany; 2 Institute of Medical Biometry and Informatics, University of Heidelberg, Heidelberg, Germany; 3 Department of General, Visceral and Transplantation Surgery, Heidelberg University Hospital, Heidelberg, Germany; 4 Department of Anesthesiology and Intensive Care Medicine, University Hospital Essen, University Duisburg-Essen, Essen, Germany

**Keywords:** Microcirculation, Haemodynamic Monitoring, Hyperspectral Imaging, Fluid Management, Tissue Water Content, Pancreatic Surgery

## Abstract

**Background:**

Hyperspectral imaging (HSI) could provide extended haemodynamic monitoring of perioperative tissue oxygenation and tissue water content to visualize effects of haemodynamic therapy and surgical trauma. The objective of this study was to assess the capacity of HSI to monitor skin microcirculation and possible relations to perioperative organ dysfunction in patients undergoing pancreatic surgery.

**Methods:**

The hyperspectral imaging TIVITA® Tissue System was used to evaluate superficial tissue oxygenation (StO2), deeper layer tissue oxygenation (near-infrared perfusion index (NPI)), haemoglobin distribution (tissue haemoglobin index (THI)) and tissue water content (tissue water index (TWI)) in 25 patients undergoing pancreatic surgery. HSI parameters were measured before induction of anaesthesia (t1), after induction of anaesthesia (t2), postoperatively before anaesthesia emergence (t3), 6 h after emergence of anaesthesia (t4) and three times daily (08:00, 14:00, 20:00 ± 1 h) at the palm and the fingertips until the second postoperative day (t5–t10). Primary outcome was the correlation of HSI with perioperative organ dysfunction assessed with the perioperative change of SOFA score.

**Results:**

Two hundred and fifty HSI measurements were performed in 25 patients. Anaesthetic induction led to a significant increase of tissue oxygenation parameters StO2 and NPI (t1–t2). StO2 and NPI decreased significantly from t2 until the end of surgery (t3). THI of the palm showed a strong correlation with haemoglobin levels preoperatively (t2**:** *r* = 0.83, *p* < 0.001) and 6 h postoperatively (t4: *r* = 0.71, *p* = 0.001) but not before anaesthesia emergence (t3: *r* = 0.35, *p* = 0.10). TWI of the palm and the fingertip rose significantly between pre- and postoperative measurements (t2–t3). Higher blood loss, syndecan level and duration of surgery were associated with a higher increase of TWI. The perioperative change of HSI parameters (∆t1–t3) did not correlate with the perioperative change of the SOFA score.

**Conclusion:**

This is the first study using HSI skin measurements to visualize tissue oxygenation and tissue water content in patients undergoing pancreatic surgery. HSI was able to measure short-term changes of tissue oxygenation during anaesthetic induction and pre- to postoperatively. TWI indicated a perioperative increase of tissue water content. Perioperative use of HSI could be a useful extension of haemodynamic monitoring to assess the microcirculatory response during haemodynamic therapy and major surgery.

**Trial registration:**

German Clinical Trial Register, DRKS00017313 on 5 June 2019

## Background

Hyperspectral imaging (HSI) combines spectroscopy with imaging and provides spatially resolved biochemical and physiologic information of imaged tissues including oxygenation, haemoglobin distribution, and water content. HSI technology enables non-contact, non-invasive real-time tissue microcirculation analysis, making it a rapidly developing imaging modality for medical applications such as image-guided surgery or wound diagnostics (Clancy et al., 2020; Lu & Fei, 2014; Saiko et al., 2020).

Perioperative haemodynamic therapy focuses on the optimization of macrocirculatory variables with the aim of maintaining an adequate supply of oxygen to tissue and organs. This essentially depends on sufficient blood flow in the smallest vessels, known as microcirculation. Severe illness or trauma can lead to an uncoupling of macro- and microcirculation, called haemodynamic incoherence (Bennett & Cecconi, 2017; Ince, 2015). In this case, the optimization of macrohaemodynamic parameters does not necessarily lead to an improvement of microcirculatory organ perfusion, contributing to detrimental fluid overload and exposure to catecholamines (Bennett & Cecconi, 2017; Malbrain et al., 2020).

In pancreatic surgery, both liberal and restrictive intraoperative fluid administration have been associated with major postoperative complications, pancreatic fistulas in particular (Andrianello et al., 2018). A liberal perioperative fluid regimen bears the risk of oedematous swelling of tissue, but a restrictive fluid administration with higher intraoperative vasopressor need could lead to impaired tissue perfusion (Bennett & Cecconi, 2017; Andrianello et al., 2018; von der Forst et al., 2020).

Bedside microcirculatory and tissue perfusion, monitoring methods are not widely incorporated into perioperative clinical practice despite technological advances and lasting research activities (Hariri et al., 2019; Huber et al., 2019). To implement holistic haemodynamic concepts that combine microcirculatory perfusion feedback with macrocirculatory resuscitation targets to resolve the risk of haemodynamic incoherence, practical bedside monitoring methods are necessary.

The TIVITA® Tissue HSI camera system (Diaspective Vision, Am Salzhaff, Germany) is a commercial product with CE (Conformité Européenne) certification suitable for clinical application. This mobile HSI camera system enables non-invasive image recording with qualitative and quantitative bedside analysis (Holmer et al., 2018; Kulcke et al., 2018; Holmer et al., 2016). It offers combined spectral and spatial evaluation of an arbitrary skin or tissue area consisting of four parameters: tissue oxygenation (StO2), near-infrared perfusion index (NPI), tissue haemoglobin index (THI) and tissue water index (TWI). Originally developed for comprehensive wound diagnostics, it has recently been evaluated for surgical applications including reconstructive and visceral surgery (Jansen-Winkeln et al., 2018; Thiem et al., 2020; Wild et al., 2018; Daeschlein et al., 2017; Kohler et al., 2019). Conceptually, this HSI system could allow monitoring of perioperative microcirculatory oxygenation and perfusion quality and tissue water content to visualize the effects of haemodynamic therapy and surgical trauma (Dietrich et al., 2020a; Dietrich et al., 2020b). The objective of this study was to assess the capacity of HSI monitoring using the TIVITA® Tissue system to identify perioperative changes of skin microcirculation and to evaluate a possible association with organ dysfunctions in patients undergoing pancreatic surgery.

## Methods

### Study design and settings

This prospective, observational pilot study was conducted by the Department of Anaesthesiology and the Department of General, Visceral and Transplantation Surgery of the Heidelberg University Hospital, Germany. The study was registered at the German Clinical Trial Register (DRKS00017313). The protocol has been published (Dietrich et al., 2020c).

### Participants

Patients undergoing elective pancreatic surgery (*n* = 25) were recruited on hospital admission and informed consent to participate was obtained. Inclusion criteria were age ≥ 18 years, signed informed consent, elective pancreatic surgery with open approach and planned postoperative admission to the intensive care unit. Exclusion criteria were pregnancy, refusal of participation and atrial fibrillation (due to the use of an uncalibrated pulse contour analysis system intraoperatively).

### HSI measurement

HSI uses the targeted light reflection and absorption of substances such as oxy-/deoxyhaemoglobin or water and enables a spatial evaluation of tissue oxygenation, perfusion quality and tissue water content (Lu & Fei, 2014). For the HSI measurements, the TIVITA® Tissue System (Diaspective Vision GmbH, Am Salzhaff, Germany) was applied. The operating principle, technical specifications and data analysis of the HSI camera system are explained in detail by Holmer et al. (Holmer et al., 2018). It offers a non-invasive, bedside evaluation of four parameters from specified wavelength ranges (Holmer et al., 2018; Kulcke et al., 2018):

- *StO2*. Tissue oxygenation (1-mm penetration depth; 500–650 nm and 700–815 nm)
- *NPI*. Near-infrared perfusion index (4–6-mm penetration depth; 655–735 nm and 825–925 nm)
- *THI*. Tissue haemoglobin index (530–590 nm and 785–825 nm)
- *TWI*. Tissue water index (880–900 nm and 955–980 nm)

StO2 is given in %. NPI, THI and TWI are index values (0–100) indicated in predefined arbitrary units. The four HSI parameters are displayed as colour-coded images. Red/yellow areas indicate high values (50–100), and green/blue areas indicate low values (0–50). HSI measurements were performed on the palm of the hand and the fingertips. The system was used according to producer operating instructions. To obtain objective numerical values, the analysis software allows the definition of regions of interest (ROI) from which the mean is calculated. The applied algorithm has been published (Holmer et al., 2018). A circular ROI was defined for each measurement site (exemplified in Fig. 2B). The ROI of the two measurement sites in this study were defined as follows:

- *Palm*. Circular area in the middle of the palm with a diameter of 70 units (the circle crossing the metacarpophalangeal joint 3–4)
- *Fingertip*. Four circular areas on the distal phalanges of finger 2–5 with a diameter of 13 units; the mean of the four values was taken

### Anaesthesia, haemodynamic management and surgical procedure

For the induction of anaesthesia, propofol and sufentanil were used. Standard muscle relaxant was rocuronium. After endotracheal intubation, an inhalational agent (sevofluran or desfluran) combined with sufentanil were used for anaesthesia maintenance. The treating anaesthesiologist determined the administration of fluids, blood products and catecholamines without a predefined goal-directed therapy algorithm. The type of pancreatic surgery was determined by the attending surgeon according to preoperative imaging and intraoperative findings.

### Measurements

**Fig. 1:**
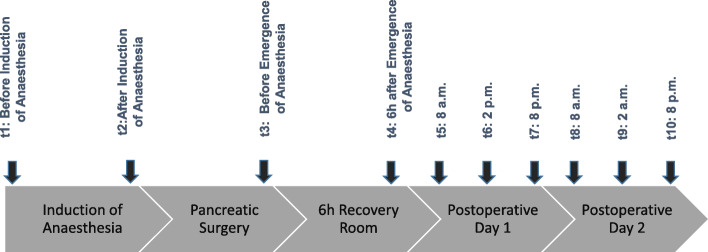
Timeline of HSI measurements during the observation period. The ten observation points are displayed on the timeline: Before induction of anaesthesia (t1); after induction of anaesthesia (t2); postoperatively before anaesthesia emergence (t3); 6 h after emergence from anaesthesia (t4); postoperative day 1, 8 a.m. (t5); postoperative day 1, 2 p.m. (t6); postoperative day 1, 8 p.m. (t7); postoperative day 2, 8 a.m. (t8); postoperative day 2, 2 p.m. (t9); and postoperative day 2, 8 p.m. (t10). HSI, hyperspectral imaging

HSI measurement results were not accessible for the treating physicians and therefore had no influence on clinical therapy. For patients undergoing pancreatic surgery, perioperative data, surgical procedure, blood loss, intraoperatively administered fluids, catecholamines, ventilator settings and anaesthetics were extracted from the clinical documentation system. HSI parameters were measured before induction of anaesthesia (t1), after induction of anaesthesia (t2), postoperatively before anaesthesia emergence (t3), 6 h after emergence from anaesthesia (t4) and three times daily (08:00, 14:00, 20:00 ± 1 h) until the second postoperative day (t5–t10) (Fig. 1). To evaluate endothelial integrity, the glycocalyx marker syndecan-1 in the serum was measured daily by ELISA (Abcam, Amsterdam, Netherlands) according to the manufacturer’s instructions. Haemodynamic parameters (e.g. cardiac index, heart rate, blood pressure) were documented simultaneously. The ProAQT system (PULSION Medical Systems SE) was used for perioperative measurements (t2 and t3) of the cardiac index by radial artery pulse contour analysis. Standard perioperative and postoperative laboratory results as well as medication, fluid balance, blood products and clinical scores were acquired from the clinical documentation system. The SOFA score was calculated preoperatively, 6 h postoperatively and once a day during the 3-day observation period.

### Primary outcome

The primary outcome parameter was the correlation of HSI parameters with perioperative organ dysfunction severity assessed with the SOFA score at the respective observation time points during the 3-day observation period.

### Secondary outcome

Secondary endpoints were the correlation of HSI parameters with vasopressor support (norepinephrine dosage [μg kg^−1^ min^−1^]), mean arterial pressure (MAP), cardiac index, lactate and glycocalyx marker (syndecan-1) levels at respective observation time points. Furthermore, the influence of intraoperative blood loss and duration of surgery on HSI parameters was assessed.

### Statistical methods

Data was collected with the aid of an electronic database system (Microsoft Excel®, Microsoft Deutschland GmbH, Unterschleißheim). SPSS (Statistical Product and Services Solutions, Version 27, SPSS Inc., Chicago, IL, USA) and Graphpad Prism (Version VIII, GraphPad Software, La Jolla, USA, Graph Pad) were used for statistical analyses. Descriptive statistics were done for the complete dataset. For continuous variables and scores, mean, standard deviation, minimum, median, quartiles and maximum were calculated and median values with interquartile range (IQR) are presented in the manuscript. Absolute and relative frequencies of categorical variables were presented. Spearman rank correlation was applied to values of HSI parameter with haemodynamic parameters and SOFA score. A Friedman test was used to analyse changes over time. A Wilcoxon test was used for the comparison of metric data between paired samples. A Mann-Whitney *U* test was used for the comparison of metric data between unpaired samples. Results of the statistical tests must be considered descriptive.

## Results

### Patient characteristics

Twenty-five patients undergoing elective major abdominal surgery due to pancreatic malignancy suspicion as well as chronic pancreatitis were included. Median age was 68 years (IQR, 15.5). Thirteen (52%) patients were male, 12 (48%) patients were female. Median weight and BMI were 68 kg (IQR, 16.5) and 24 kg/m^2^ (IQR, 3), respectively. Patients in this study had a light or slightly tanned skin type. The median Revised Cardiac Risk Index was 1 (IQR, 1) and all patients were classified in the third category of the classification of the American Society of Anaesthesiologists (ASA 3). Underlying aetiologies were pancreatic cancer (*n* = 20, 80%), chronic pancreatitis (*n* = 3, 12%), liposarcoma (*n* = 1, 4%) and neuroendocrine tumour of the pancreatic gland (*n* = 1, 4%). Performed operative procedures included partial pancreatoduodenectomy (*n* = 14, 56%), total pancreatectomy (*n* = 5, 20%) and multivisceral resection including distal pancreatectomy (*n* = 1, 4%). In 5 patients (20%), pancreatic biopsies were performed without major resection due to advanced tumour disease. The median operation duration was 305 min (IQR, 157) and the median intraoperative blood loss was 800 ml (IQR, 1.125 ml). Two (8%) patients underwent relaparotomy due to a 2-step surgical procedure and an insufficiency of the biliodigestive anastomosis. Two (8%) patients developed a pancreatic fistula type B. No patient died within 30 days postoperatively.

### Analysis of perioperative HSI parameters

**Fig. 2:**
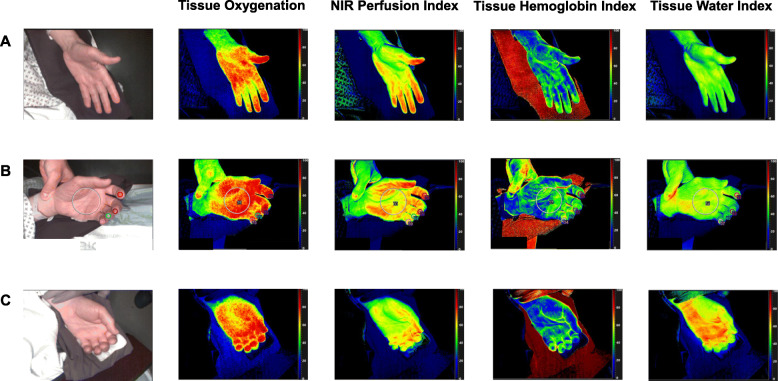
HSI images of the right palm and fingertip (finger II–V) of patient undergoing pancreatic surgery. **A** and **B** were recorded before and after induction of anaesthesia, respectively. **C** was taken postoperative before anaesthesia emergence. Tissue oxygenation (StO2), near-infrared perfusion index (NPI), tissue haemoglobin index (THI) and tissue water index (TWI) are displayed colour-coded (scale at the right side of each image). Red/yellow areas indicate high values (50–100), and green/blue (0–50) areas indicate low values. The numerical scale ranges from 0 to 100. The regions of interest are exemplarily drawn in the images of B. StO2 is given in % and THI, NPI and TWI are index values in arbitrary units. HSI, hyperspectral imaging

**Fig. 3:**
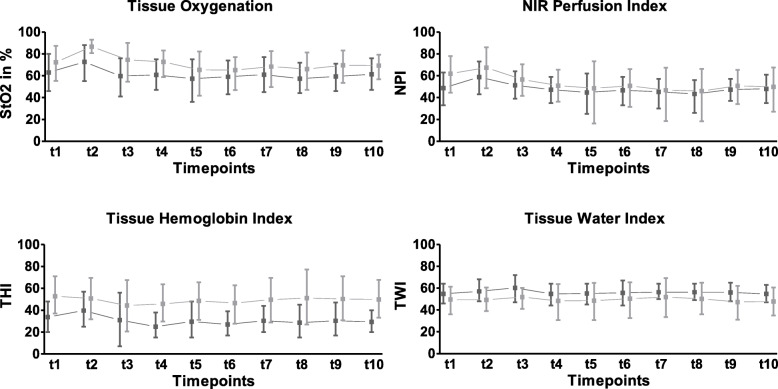
The perioperative course of HSI parameters of the palm (dark grey) and the fingertip (light grey) in patients undergoing pancreatic surgery; Tissue oxygenation (StO2), NIR perfusion index tissue (NPI), haemoglobin index (THI) and tissue water index (TWI) values are presented as grey squares at the observation timepoints (t1–t10; *x*-axis) as median value with interquartile range. The numerical scale ranges from 0 to 100 (*y*-axis). StO2 is given in and NPI, THI and TWI are index values in arbitrary units. HSI, hyperspectral imaging

Two hundred and fifty HSI measurements on 25 patients undergoing pancreatic surgery were analysed. Exemplary hyperspectral images of one patient before induction of anaesthesia (Fig. 2A), after induction of anaesthesia (Fig. 2B) and after surgery before anaesthesia emergence (Fig. 2C) are shown in Fig. 2. The perioperative courses of the HSI parameters of the palm and the fingertip are displayed in Fig. 3. Additionally, we examined perioperative factors that could have influenced the observed HSI parameters including surgical conditions (blood loss, surgery duration, haemoglobin level), haemodynamic parameters (cardiac index, MAP, lactate level), haemodynamic management (norepinephrine dose, fluid balance) and one glycocalyx marker (syndecan-1 level) (Table 1). Finally, we analysed possible correlations of HSI measurements with perioperative changes in organ dysfunction assessed by SOFA score.

**Table 1:** The course of plasma syndecan level, cardiac index, mean arterial pressure, temperature, haemoglobin level, vasopressor dose, lactate level, fluid balance and SOFA score. The values are presented as median with interquartile range. Friedman test was used to analyse changes over time

	T1	T2	T3	T4	T5	T6	T7	T8	T9	T10	*p*-value
**Syndecan level (ng/ml), n**	-	47.0 (43), 25	79.5 (90), 25	-	67.4 (86), 25	-	-	61.1 (44), 25	-	-	0.0008
**Cardiac index (l/min/m**^**2**^**), n**	-	2.1 (0.8), 25	3.3 (0.9), 25	3.3 (1.0), 15	-	-	-	-	-	-	0.03
**MAP (mmHg), n**	109 (17), 25	78 (14), 25	80 (24), 25	77 (24.5), 25	76 (23), 25	75 (19), 25	83 (25.5), 25	82 (23), 25	84 (20.5), 25	83 (19), 25	< .0001
**Temperature (°C), n**	36,0 (1), 25	36,0 (0.7), 25	36,6 (0.6), 25	36,9 (0.7), 25	37,0 (0.6), 25	37,0 (0.4), 25	37,2 (0.3), 25	36,9 (0.3), 25	36,8 (0.5), 25	36,9 (0.6), 25	< .0001
**Haemoglobin (g/dl), n**	12.8 (1.8), 25	12.0 (1.7) , 24	12.2 (2.9), 24	11.4 (2.9), 18	9.8 (2.6), 24	9.9 (2.0), 10	9.5 (1.9), 10	8.6 (1.8), 17	9.8 (1.5), 8	9.3 (2.0), 9	< .0001
**Norepinephrine dose (μg/kg/min), n**	0.0 (0), 25	0.0 (0), 25	0.04 (0.07), 25	0.0 (0), 25	0.00 (0), 25	0.0 (0), 25	0.0 (0), 25	0.0 (0), 25	0.0 (0), 25	0.0 (0), 25	< .0001
**Lactate level (mg/dl), n**	-	8.0 (1.7), 20	10.2 (4.7), 24	11.5 (5.4), 18	14.7 (7.6), 13	15.4 (12.2), 9	16.4 (9.3), 9	11.3 (5.7), 9	10.9 (4.0), 8	10.0 (3.7), 9	0.0001
**Fluid balance (ml), n**	-	0, 25	1700 (750), 25	2560 (1233), 25	2399 (2140), 25	2452 (1787), 25	2640 (1576), 25	2536 (2172), 22	2478 (2602), 22	2551 (3898), 22	
**SOFA score, n**	0 (1), 25	-	-	3 (3), 25	2 (2), 25	-	-	1 (2), 22	-	-	0.0012

*MAP* mean arterial pressure, *n* number of available patients, *SOFA score* sequential organ failure assessment score

### Anaesthesia induction resulted in an increase of skin HSI parameters whereas mean arterial pressure and heart rate decreased

Anaesthesia induction (t1–t2) resulted in a significant increase of palm StO2 (t1, 63% (IQR, 14) vs. t2, 75% (IQR, 12); *p* < 0.001) and fingertip StO2 (t1, 75% (IQR, 10) vs. t2, 86% (IQR, 7); *p* < 0.001). At the palm, NPI rose significantly (t1, 50 (IQR, 10) vs. t2, 60 (IQR, 12); *p* < 0.001), but not at the fingertip (t1, 63 (IQR, 13) vs. t2, 68 (IQR, 9); *p* = 0.115). Palm THI (t1, 33 (IQR, 12) vs. t2, 37 (IQR, 11); *p* < 0.001) and palm TWI (t1, 54 (IQR, 5) vs. t2, 55 (IQR, 6); *p* = 0.035) showed a significant increase during anaesthetic induction. At the fingertip, both THI (t1, 51 (IQR, 14) vs. t2, 51 (IQR, 14); *p* = 0.851) and TWI (t1, 52 (IQR, 10) vs. t2, 49 (IQR, 10); *p* = 0.201) did not change significantly. Mean arterial pressure (MAP) (t1, 109 mmHg (IQR, 17) vs. t2, 78 mmHg (IQR, 14); *p* < 0.001) and heart rate (HR) (t1, 76/min (IQR, 26) vs. t2, 54/min (IQR, 14); *p* <0.001) decreased during anaesthesia induction (t1–t2).

### At the end of surgery, HSI measurements showed a decrease in tissue oxygenation and tissue haemoglobin index paralleled by an increase in tissue water content

StO2 of the palm (t2, 75% (IQR, 12) vs. t3, 62% (IQR, 15); *p* <0.001) and the fingertip (t2, 86% (IQR, 7) vs. t3, 79 % (IQR, 12); *p* < 0.001) both decreased significantly from the beginning (t2) to the end of surgery (t3). Correspondingly, NPI showed a reduction at the palm (t2, 60 (IQR, 12) vs. t3, 51 (IQR, 10); *p* < 0.001) and the fingertip (t2, 68 (IQR, 9) vs. t3, 58 (IQR, 9); *p* < 0.001). THI of the palm (t2, 37 (IQR, 11) vs. t3, 30 (IQR, 14); *p* = 0.001) and the fingertip (t2, 51 (IQR, 14) vs. t3, 45 (IQR, 12); *p* = 0.025) decreased during the operative period. TWI of the palm (t2, 55 (IQR, 6) vs. t3, 62 (IQR, 7); *p* = 0.003) and TWI of the fingertip (t2, 49 (IQR, 10) vs. t3, 54 (IQR, 8); *p* = 0.031) increased significantly from the beginning to the end of surgery. MAP did not differ between t2 and t3 (t2, 78 mmHg (IQR, 14) vs. t3, 80 mmHg (IQR, 24); *p* = 0.119). HR was increased at the end of surgery (t2, 54/min (IQR, 14) vs. 76/min (IQR, 24); *p* < 0.001). Cardiac index (t2, 2.1 l/min/m^2^ (IQR, 0.8) vs. t3, 3.3 l/min/m^2^ (IQR, 0.9); *p* = 0.001) increased significantly during the operation.

### Tissue oxygenation HSI parameters correlated with perioperative lactate changes whereas no correlation to macrohaemodynamic variables was observed

The perioperative changes (∆t2–t3) of lactate levels and the HSI tissue oxygenation parameters StO2 (*r* = − 0.46, *p* = 0.048) and NPI (*r* = − 0.51, *p* = 0.026) of the fingertip, were moderately negatively correlated. Conversely, THI of the fingertip (*r* = 0.59, *p* = 0.008) and palm (*r* = 0.54, *p* = 0.017) was positively correlated to perioperative changes in lactate levels (∆t2–t3). Intraoperative MAP, cardiac index measurements and norepinephrine dose showed no correlation with the HSI tissue oxygenation parameters StO2 and NPI.

### Tissue haemoglobin index correlated with haemoglobin level at individual measurement timepoints during the observation period

THI of the palm showed a strong correlation with haemoglobin levels preoperatively (t2: *r* = 0.83, *p* < 0.001) and 6 h postoperatively (t4: *r* = 0.71, *p* = 0.001). No significant correlation was observed between haemoglobin levels and palm THI at the end of surgery (t3: *r* = 0.35, *p* = 0.10).

### Tissue oxygenation HSI parameters did not differ between patients with higher and lower blood loss

**Fig. 4:**
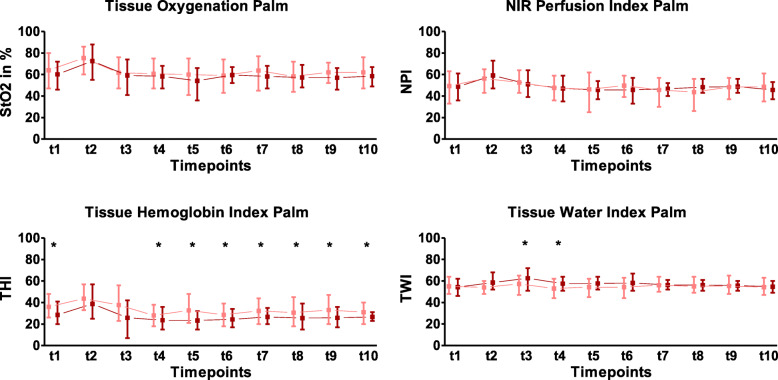
The perioperative course of HSI parameters of the palm in patients undergoing pancreatic surgery with high (> 800 ml, dark red) and low (≤ 800 ml, light red) intraoperative blood loss; Tissue oxygenation (StO2), near-infrared perfusion index (NPI), tissue haemoglobin index (THI) and tissue water index (TWI) values of patients with high and low intraoperative blood loss are presented as dark red and light red squares at the observation timepoints (t1–t10) as median value with interquartile range, respectively. The numerical scale ranges from 0 to 100 (*y*-axis). StO2 is given in % and NPI, THI and TWI are index values in arbitrary unit. Statistical analysis was performed with the Mann-Whitney *U* test. *p < 0.05; HSI, hyperspectral imaging

Next, we divided the patients into two groups based on the median intraoperative blood loss (800 ml; IQR, 1.125 ml) in a higher blood loss (> 800 ml) and a lower blood loss (≤ 800 ml) group. The course of palm HSI parameters in the two blood loss groups is shown in Fig. 4. We did not observe a difference in palm StO_2_ and NPI between the two blood loss groups (Fig. 4).

### Higher blood loss is associated with increased palm tissue water content at the end of surgery

Palm TWI did not differ between the two blood loss groups before the beginning of surgery (t1–t2) (Fig. 4). Patients with higher blood loss showed a significantly higher palm TWI at the end of surgery (t3, 64.5 (IQR, 6.3) vs 60.0 (IQR, 3.5), *p* = 0.012) and 6 h after anaesthesia emergence (t4, 57.5 (IQR, 6) vs 53.0 (IQR, 6), *p* = 0.018) compared to patients with lower blood loss. On the following 2 postoperative days, no difference in palm TWI was observed between the blood loss groups (t5–t10) (Fig. 4).

### Palm tissue haemoglobin index discriminated between patients with higher and lower intraoperative blood loss in the postoperative observation time

Patients with higher intraoperative blood loss showed a lower palm THI compared to the lower blood loss group starting 6 h postoperatively (t4, 20.0 (IQR, 3.8) vs 28.0 (IQR, 9.5), *p* = 0.012). This difference in palm THI remained significant until the end of the observation period (t5–t10). Of note, patients with higher blood loss demonstrated a lower palm THI before anaesthesia induction (t1, 24.5 (IQR, 11.75) vs. 34.0 (IQR, 7.5), *p* = 0.024) (Fig. 4).

### The duration of surgery, intraoperative fluid balance and the change of syndecan-1 level correlated with HSI measured tissue water content at the end of surgery

Further analysis revealed a strong correlation between the duration of surgery and palm TWI at the end of surgery (t3: *r* = 0.61, *p* = 0.001). Fluid balance at the end of surgery correlated weakly with palm (t3: *r* = 0.43, *p* = 0.03) and fingertip TWI (*r* = 0.41, *P* = 0.04). The change in plasma syndecan levels (t2–t3) showed a moderate correlation with the change of palm TWI (t2–t3) (*r* = 0.5, *p* = 0.01). Palm TWI showed a moderate positive correlation with syndecan levels on the first (t5: *r* = 0.52, *p* = 0.007) and the second (t8: *r* = 0.5, *p* = 0.01) postoperative day.

### HSI parameters did not correlate with perioperative organ dysfunction

The median initial (t1) SOFA score was 0 points (IQR, 1) and increased up to 3 points (IQR, 3.5) at 6 h after emergence from anaesthesia (t4). On the first and second postoperative day, the median SOFA score decreased to 2 (IQR, 2) and 1 point (IQR, 2.25), respectively. The perioperative change of hyperspectral imaging parameters (∆t1–t3) did not correlate with the perioperative change of the SOFA score. In 16 (64%) patients, the SOFA score increased perioperatively. At t4, the HSI parameters in these patients did not differ from patients without a perioperative increase in SOFA score. Due to the small sample size and the low number of postoperative complications, we were not able to assess the relation of HSI parameters to perioperative complications or mortality.

## Discussion

The present study is the first explorative evaluation using the TIVITA® Tissue System for perioperative hyperspectral imaging for skin microcirculatory assessment during major abdominal surgery. Haemodynamic therapy aims to maintain tissue perfusion and oxygen delivery to protect organ function during surgery. Alterations in skin microcirculation are associated with organ dysfunction, morbidity and mortality in several cohorts of surgical and critically ill patients (Hariri et al., 2019; van Genderen et al., 2014; Jhanji et al., 2009). Non-invasive bedside technology that recognizes tissue oxygenation abnormalities and potentially alerts physicians of detrimental fluid and vasopressor effects is therefore envisioned to improve clinical outcomes in major surgery. Various technical devices, which allow an objective measurement of microcirculation, are the focus of clinical research, but so far, no technology has been broadly applied in perioperative practice (Huber et al., 2019). HSI is a non-invasive imaging technology that provides diagnostic information about biochemical tissue characteristics including oxygenation, haemoglobin, and water content (Liu et al., 2014). The parallel assessment of the TIVITA® Tissue System’s HSI parameters StO2, NPI and THI enables a spatial indication of oxygenation and perfusion quality including differentiated information about superficial (StO2) and deeper tissue layer (NPI) oxygenation (Holmer et al., 2018; Kulcke et al., 2018).

Induction of anaesthesia led to a distinct increase in StO2 and NPI despite a concomitant decrease of MAP. Propofol has vasodilative effects explaining the drop of blood pressure and the observed increase in peripheral oxygenation and perfusion quality. Our observations during anaesthesia induction demonstrate that clinically relevant short-term changes in skin microcirculation can be measured with HSI. This finding is in line with previous results regarding the measurement capabilities of palm StO2, NPI, and THI during venous and arterial occlusion tests (Holmer et al., 2018).

Haemodynamic incoherence is characterized by persisting microcirculatory alterations despite restoration of macrohaemodynamic targets and is associated with detrimental effects of fluid and vasopressor therapy. Haemodynamic incoherence is a frequent phenomenon in a state of critical illness or trauma (Ince & Ertmer, 2016). In the present study, cardiac index increased, and MAP was similar between the pre- and postoperative measurements. However, the microcirculatory tissue oxygenation parameters StO2 and NPI decreased during surgery contrasting the observed macrohaemodynamic parameters. Of note, perioperative lactate dynamics correlated with fingertip StO2 and NPI as well as fingertip and palm THI suggesting that these HSI parameters might be indicative for the presence of systemic oxygenation deficits despite stable macrohaemodynamic parameters.

Conceptually, macrohaemodynamic guided therapy should be accompanied by the evaluation of microcirculatory parameters to identify deficits in tissue oxygenation to prevent organ damage and perioperative complications. Skin perfusion abnormalities have been associated with the development of severe complications independently of macrohaemodynamics following major abdominal surgery (van Genderen et al., 2014). In a study on perioperative sidestream darkfield imaging in gastrointestinal surgery, patients with or without complications did not differ in terms of macrohaemodynamic parameters. However, retrospectively, patients with a perioperative adverse event showed reduced microcirculatory perfusion parameters pre- and postoperatively (Jhanji et al., 2010). In critical care, Kazune et al. used HSI to assess skin tissue oxygenation in septic patients and showed that skin microcirculation could be influenced by haemodynamic therapy. Raising the MAP by 20 mmHg, skin perfusion parameters improved significantly (Kazune et al., 2019a). This indicates that skin microcirculatory monitoring via HSI could provide the possibility of evaluating the effectiveness of haemodynamic therapy based on its effects on tissue oxygenation. In a further analysis of septic patients, Kazune et al. showed a correlation between organ dysfunction and skin hyperspectral imaging parameters (Kazune et al., 2019a; Kazune et al., 2019b). The present study did not show a correlation between perioperative organ dysfunction assessed by SOFA score and HSI parameters. Due to the low number of postoperative complications in our small study cohort, it was not possible to investigate any association of HSI parameters with perioperative morbidity and mortality. A possible explanation for our results is that the patients did not experience an intraoperative circulatory compromise that might have inflicted persistent postoperative organ dysfunction. Furthermore, the transient postoperative organ dysfunction during the observation period points to a minor effect of surgical trauma on overall organ function. Vulnerability to microcirculatory dysfunction due to impaired perfusion and tissue oxygenation varies between different vascular beds and organ systems. An important research question is whether skin HSI monitoring data reflect concomitant systemic or organ-specific microcirculatory compromise and/or organ dysfunction. Recent data on skin HSI monitoring in septic patients points to an association with organ dysfunction (Kazune et al., 2019a; Kazune et al., 2019b; Dietrich et al., 2021). Surgical data showed that relevant intraoperative changes in organ oxygenation and perfusion quality can be identified by HSI monitoring (Jansen-Winkeln et al., 2018; Kohler et al., 2019; Thiem et al., 2021). Future studies should therefore examine the relationship between HSI monitoring data and the function of different organ systems during haemodynamic compromise to answer whether skin HSI indicates coherent microcirculatory impairment of multiple organ systems. Our group previously showed that palm and fingertip HSI evaluation in sepsis patients revealed a distinctive pattern of reduced skin oxygenation and perfusion quality in combination with microcirculatory blood pooling and increased tissue water content (Dietrich et al., 2021). Although we did not observe a correlation with perioperative organ dysfunction in patients with major abdominal surgery, the results of this study add new aspects regarding bedside HSI as microcirculatory monitoring device.

In the present study, we found a strong correlation of THI with preoperative and 6 h postoperative haemoglobin levels, but there was no significant correlation at the end of surgery. Palm and fingertip THI decreased significantly during the operative period and showed a positive correlation to changes in lactate levels. This indicates the influence of factors other than the haemoglobin level on the THI. Perfusion changes, haemodilution due to fluid therapy and blood loss are factors that possibly influence skin THI during surgery. In a surgical study on flap monitoring, a low THI was a sign of reduced perfusion caused by arterial occlusion. In contrast, an increase in THI was observed in cases of venous congestion (Thiem et al., 2021). We proposed that an elevated THI might reflect pronounced alteration of skin microcirculation indicative for disturbed perfusion and a stagnant flow situation in septic patients. Furthermore, palm and fingertip THI discriminated between non-survivors and survivors and was predictive for 28-day survival in sepsis patients (Dietrich et al., 2021). In the present study, postoperative THI values discriminated between patients with higher and lower blood loss. The finding of lower preoperative palm THI values in patients with higher blood loss was unexpected and cannot be conclusively explained now. These results point to risk-stratification properties of THI in surgical patients at risk to develop sequel complications of major bleeding and need further examination in follow-up studies.

Surgical morbidity and mortality are associated with intraoperative and postoperative fluid management (Bennett & Cecconi, 2017). Both fluid overload and hypovolemia can have detrimental effects on perioperative morbidity (Malbrain et al., 2020; von der Forst et al., 2020). There is no established bedside method to measure changes in tissue water content in perioperative and critical care medicine. HSI technology allows real-time visualization and evaluation of tissue water content based on the specific light absorption characteristics of water (Holmer et al., 2018; Liu et al., 2014). We observed a significant increase in palm and fingertip TWI from the beginning to the end of surgery in this study. We evaluated syndecan levels in this study as a read out for perioperative vascular glycocalyx shedding. Vascular glycocalyx shedding is a well-known consequence of both surgical trauma as well as fluid administration promoting vascular leakage and extravasation of fluid into tissue (Astapenko et al., 2019). The correlations of perioperative fluid balance, syndecan levels, higher blood loss, and surgery duration with skin TWI indicate that fluid management and surgical trauma contribute to the increased TWI during surgery. Patients with higher blood loss demonstrated significantly higher palm TWI at the end of surgery and 6 h after anaesthesia emergence suggesting a pronounced vascular leakage in these patients. This effect did not continue in the postoperative period indicating restoration of the vascular barrier function and reabsorption of the increased tissue water content. Our finding concurs with wound care management results using longitudinal skin HSI showing that a decrease in TWI indicative of a reduction in wound oedema (Daeschlein et al., 2017). Fluid administration is a cornerstone of haemodynamic therapy but carries the risk of detrimental fluid overload threatening surgical outcome. In septic patients, a significantly increased palm and fingertip TWI could be observed compared to healthy controls. Palm TWI during sepsis was persistently increased and correlated positively with the SOFA score on the third day following ICU admission (Dietrich et al., 2021). These results point to the potential of perioperative bedside HSI monitoring to detect increased skin water content and provide real-time stop signs to avoid fluid overload and tissue oedema.

HSI technologies are increasingly becoming the subject of surgical research for real-time intraoperative organ perfusion quality evaluation, resection planning, and anastomosis optimization (Kohler et al., 2019; Sucher et al., 2019; Barberio et al., 2020). A general short coming of microcirculatory monitoring approaches is that microcirculatory alterations in one organ do not necessarily reflect the situation in other organs. Therefore, we propose combined intraoperative skin and organ HSI measurements in the surgical field during abdominal surgery. This approach could open the possibility of interdisciplinary tissue perfusion-guided haemodynamic monitoring together with optimized surgical results.

Our study has several limitations: the TIVITA® Tissue system’s novel technical specifications limit the overall comparability of our results to alternative HSI technologies and other established optical microcirculatory monitoring technologies or clinical evaluation methods for peripheral perfusion. We decided not to compare the HSI measurements to established microcirculatory monitoring methods in this first exploratory study. We did not perform intraoperative organ-specific HSI measurements to avoid impairment of the surgical procedure. Therefore, we cannot report if HSI changes in skin microcirculation were paralleled by changes in other organ systems. In this regard, the use of the perioperative SOFA score as an endpoint to assess organ dysfunction needs to be carefully considered in data interpretation. SOFA is a composite score reflecting the severity of organ dysfunction in six organ systems. Currently, knowledge about correlations of HSI data and organ-specific changes affecting SOFA categories is limited. We decided to use SOFA scoring to evaluate organ dysfunction in this study because previous studies showed an association between skin HSI measurements and SOFA score in critically ill septic patients (Kazune et al., 2019a; Kazune et al., 2019b; Dietrich et al., 2021). Therefore, consistent use of SOFA scoring at least provides a basis for interpreting the emerging HSI data for microcirculatory monitoring in different cohorts of critically ill patients such as surgical and septic patients. A general limitation of HSI technologies is that highly pigmented skin areas cannot be measured reliably due to melanin — all patients in this study had a light or slightly tanned skin type. The final extent of the surgical procedure was determined intraoperatively leading to heterogeneity in the performed surgical procedures. The small sample size of this exploratory study must be considered carefully in the interpretation of our results. All analyses are of explorative nature and *p*-values ≤ 0.05 were termed significant for descriptive reasons only.

## Conclusion

This is the first study using HSI to visualize changes in skin oxygenation parameters together with tissue water content in patients undergoing pancreatic surgery. HSI was able to measure perioperative changes of superficial and deeper layer tissue oxygenation and perfusion quality. HSI could provide new insight for perioperative fluid therapy by visualizing tissue water content. However, a direct linkage to postoperative organ dysfunction and complications needs to be investigated in further studies. Bedside HSI could enrich future perioperative haemodynamic monitoring and open up possibilities for tissue oxygenation and perfusion-guided therapy.

## Data Availability

The datasets used and analysed during the current study are available from the corresponding author on reasonable request.

## Abbreviations

HR: Heart rate
HSI: Hyperspectral imaging
IQR: Interquartile range
MAP: Mean arterial pressure
*n*: Number of patients
NPI: Near-infrared perfusion index
*r*: Coefficient of correlation
ROI: Region of interest
SOFA score: Sepsis-related Organ Failure score
StO2: Tissue oxygenation
t: Point in time
THI: Tissue haemoglobin index
TWI: Tissue water index
vs.: Versus
